# Eosinophilic Myocarditis: An Often-Overlooked Diagnosis in Patients Presenting with Heart Failure

**DOI:** 10.1155/2022/8453581

**Published:** 2022-07-01

**Authors:** Young Min Cho, Abdullah Asreb, Riaz Mahmood, Ahmad Moaz, Ugochukwu Egolum

**Affiliations:** ^1^Internal Medicine Residency Program, Northeast Georgia Medical Center, Gainesville, Georgia, USA; ^2^Georgia Heart Institute, Northeast Georgia Medical Center, Gainesville, Georgia, USA

## Abstract

*Introduction*. Hypereosinophilic syndrome (HES) is a rare disease characterized by unexplained peripheral eosinophilia along with evidence of end-organ damage. Cardiac involvement is the most life-threatening consequence and is frequently underreported with a prevalence of around 5%. The gold standard for diagnosis is myocardial biopsy, but less-invasive imaging such as cardiac MR (CMR) has been frequently used to help with the diagnosis. We are presenting a unique case of a patient diagnosed with Eosinophilic myocarditis (EM) supported by CMR with rapid improvement after starting steroid treatment. *Case Presentation*. A 67-year-old African American female with extensive cardiovascular disease history presenting with chest pain was diagnosed with EM secondary to hypereosinophilic syndrome (HES). Lab workup revealed absolute eosinophils of 4.70 × 10^3^/*μ*L (normal 0–0.75 × 10^3^/*μ*L). Transthoracic echocardiography showed mild reduction in left ventricular function and a large obliterating thrombus in the right ventricular apex. CMR showed increased signal intensity at the left ventricular and right ventricular apex, consistent with myocardial edema. Subsequently, the patient was placed on dexamethasone 10 mg daily with significant symptomatic improvement. *Discussion*. EM is a rare complication of hypereosinophilic syndrome and can mimic common cardiovascular diseases such as acute exacerbation of heart failure or myocardial infarction. A high index of suspicion is essential especially in the setting of suggestive lab workup. CMR is a promising noninvasive and cost-effective alternative for myocardial biopsy in diagnosis.

## 1. Introduction

Hypereosinophilic syndrome (HES) is a rare disease characterized by unexplained peripheral eosinophilia along with evidence of end-organ damage. The estimated prevalence is between 0.36 and 6.3 per 100,000 [1]. It is defined as an absolute eosinophilic count (AEC) of >1.5 × 10^3^ cells/microL (*μ*L) in the peripheral blood on two examinations separated in time by at least one month and/or pathologic evidence confirming tissue hypereosinophilia (HE) [2]. The persistent hypereosinophilia leads to eosinophilic-mediated organ dysfunction, and cardiac manifestations occur in only about 5% of the cases [3]. Earlier studies described much lower prevalence of cardiac involvement, but with the advancements in imaging and biopsy, the diagnosis rate is gradually increasing [4] [5]. As described by Loeffler in 1973, eosinophil-mediated heart disease has three stages [6]. The first stage is characterized by acute early necrosis due to eosinophilic and lymphocytic infiltration followed by subsequent eosinophil degranulation and microabscess formation within the myocardium [7]. In the second stage, thrombi formation occurs along the damaged endocardium of either or both ventricles and occasionally even within the atrium [8]. The final stage is the fibrotic stage, where the thrombus converts to fibrosis. This process leads to progressive tissue scarring and ultimately restrictive cardiomyopathy due to eosinophilic myocarditis (EM). Common clinical presentations are dyspnea, chest pain, signs of left-sided and/or right-sided congestive heart failure (CHF), mitral regurgitation, cardiomegaly, and T wave inversions. To this date, there are no standardized diagnostic criteria for EM, and typically, a multimodal imaging approach is utilized. Electrocardiogram (EKG) manifestations of myocarditis are ST-T changes, atrioventicular block (AV), bundle branch block, and ventricular arrhythmias, but the sensitivity and specificity are not enough to diagnose EM [9]. Transthoracic echocardiography (TTE) can detect intracardiac thrombi. The extent of fibrosis shows thickening of involved valve leaflets, and an increase in the intensity of endomyocardial echoes in areas of endomyocardial fibrosis. However, the accuracy of TTE to diagnosis is limited due to highly variable finding associated with myocarditis resulting false negative diagnosis [10]. Coronary angiography does not diagnose the EM, but it helps to rule out acute coronary disease. Cardiac magnetic resonance imaging (CMR) imaging plays a major role in the diagnosis as it reliably detects all aspects and stages of eosinophilic-mediated heart damage. The pattern of cardiac manifestations is variable and can include myocardial edema, fibrosis, and sedimentation of thrombotic material and reduced systolic or diastolic function [11]. Cardiac biopsy provides definitive evidence of eosinophil-associated cardiac disease by showing infiltration with eosinophils by staining of the biopsy for antibodies for eosinophil granule proteins [12]. Biopsy is usually reserved for cases where cardiac eosinophil involvement is uncertain. Cardiac MRI has high sensitivity for even the earliest stages of EM, and in this case report, we will highlight the role of CMR as a noninvasive tool for the early diagnosis of EM.

## 2. Case Presentation

**Figure 1:**
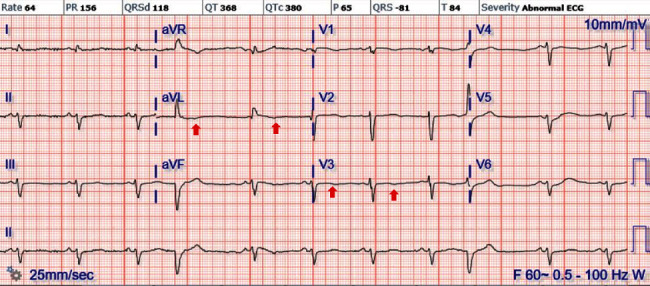
Electrocardiogram on a day of admission with sinus rhythm, left axis deviation, T wave inversion in aVL, and V3 and multiple premature ventricular contraction.

**Figure 2:**
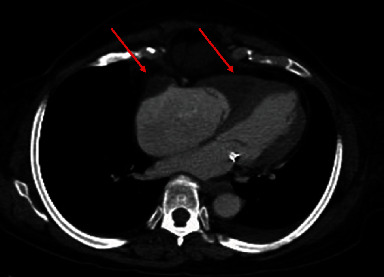
CTA showing evidence of right atrial and right ventricular apical thrombus (red arrows).

**Figure 3:**
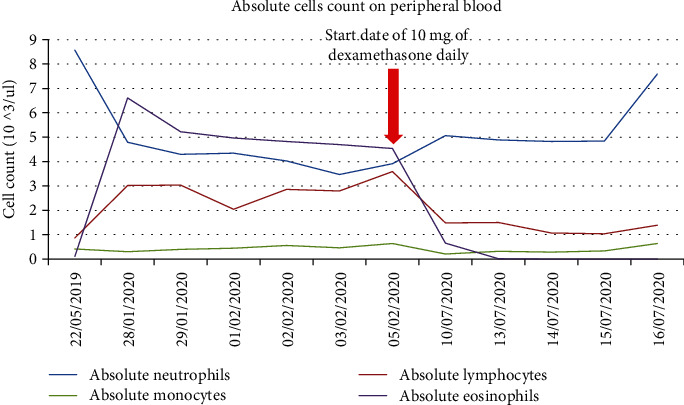
Trend of absolute eosinophil count from 5/22/19 to 7/16/2020 and start date of dexamethasone 10 mg daily.

**Figure 4:**
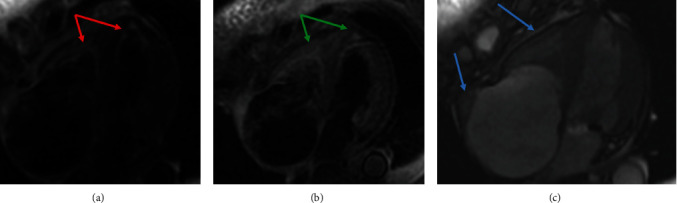
CMR. (a) Double inversion recovery (IR) T2-weighted axial imaging showing increased signal intensity of myocardium in the appendix of left and right ventricles consistent with myocardial edema (red arrows). (b) Tripe IR T1-weighted fat saturation axial image showing again signal intensity in the myocardium (green arrows). (c) Balanced steady state free precession (bSSFP) horizontal long axis still frame showing increase signaling with severe right atrial enlargement and possible thrombus (blue arrows).

A 67-year-old African American female with an extensive history of cardiovascular disease including heart failure with preserved ejection fraction (EF of 50-55%), hypertension, coronary artery disease treated with coronary artery bypass graft surgery, mitral valve repair, stage IV chronic kidney disease, and dyslipidemia presented to the ED with chest pain and interscapular back pain for four days. The chest pain was intermittent, sharp, and worsened with deep inspiration. On presentation, she was afebrile, with a blood pressure of 121/86 mmHg, a heart rate of 70 bpm, and a respiratory rate of 20 breaths per minute with SpO2 of 100% on room air. Physical examination was notable for jugular venous distension (JVD) at more than 10 cm H_2_O at 90^o^. Laboratory workup was remarkable (creatinine level of 2.2 mg/dL (NR 0.6-1.00 mg/dL), BUN 31 mg/dL (NR 3.0-23.0 mg/dL), hemoglobin (Hgb) 9.8 g/dL (NR 12.0-16.0 g/dL), hematocrit 30.6% (NR 37.0-47.0%), absolute eosinophils 4.70 × 10^3^/*μ*L (NR 0–0.75 × 10^3^/*μ*L), relative eosinophils 49% (NR 0-7%), D-dimer 3.118 *μ*g/mL FEU (NR ≤ 0.400 *μ*g/mL FEU), and B-type natriuretic peptide (BPEP) of 1,385.2 pg/mL (NR 0.0–100.0 pg/mL)). Cardiac panel was performed every 3 hours for ischemic evaluation and consistently remained within normal limits. Electrocardiogram (EKG) showed sinus rhythm, left axis deviation, T wave inversion in aVL, and V1, multiple premature ventricular complexes, and left anterior fascicular block (Figure 1). Her chest X-ray was unremarkable for acute finding. Chest computed tomography angiography (CTA) was negative for pulmonary embolism or aortic dissection; however, there was evidence of right atrial and right ventricular apical thrombus (Figure 2). Transthoracic echocardiography (TTE) demonstrated midrange left ventricular systolic function (estimated ejection fraction 45-50%). Right ventricular systolic function was moderately reduced. A homogeneous obliterative mass was detected at the right ventricular apex (video 1). Subsequently, the patient was placed on unfractionated heparin infusion. A review of her medical records revealed a high level of eosinophils for the past 3 months (Figure 3), qualifying her for a diagnosis of HES. Her eosinophilia in combination with the imaging findings was highly suggestive of eosinophilic myocarditis (EM). Cardiac MRI without gadolinium contrast infusion due to worsening renal function was subsequently performed. T2-weighted images showed increased signal intensity at the left ventricular and right ventricular apex, consistent with myocardial edema (MRI Figure 4(a)). T1-weighted images showed increased signal intensity in the same area (MRI Figure 4(b)). Balanced steady free precession images showed mildly reduced left ventricular systolic function with calculated EF of 44% with probable right ventricular apical and right atrial thrombus (MRI Figures 4(c)). Clinical history in combination with imaging data from echocardiography and CMR was highly suggestive of eosinophilic myocarditis. Further studies including SARS-COV-2 PCR and Stool ova/parasite studies were negative. Laboratory workup for antinuclear antibodies (ANA), anti-DNA, perinuclear antineutrophil cytoplasmic antibodies (p-ANCA), and antimyeloperoxidase (MPO) were also negative. Bone marrow biopsy was performed and showed hypereosinophilia. Fluorescence in situ hybridization (FISH) for factor interacting with PAPOLA and CPSF1 (FIP1L1) platelet-derived growth factor receptor *α* (PDGFRA) was negative for rearrangements. Given her presentation and workup suggestive of HES, the patient received the diagnosis of EM due to HES. The patient was started on corticosteroids with significant improvement in her symptoms. With the patient's improvement after steroids and highly suggestive findings on MRI, cardiac biopsy was deferred. She was discharged home on oral anticoagulation and steroids with close follow-up with cardiology.

## 3. Discussion

HES is a rare disease which must be suspected in individuals with persistent eosinophilia in peripheral blood on at least two occasions with a minimum time interval of 4 weeks [3]. The diagnosis of EM is challenging particularly due to the lack of specific diagnostic criteria and rarity of the condition. A high index of suspicion must be kept while working up patients with peripheral eosinophilia and heart failure. During the diagnostic workup, it is crucial also to identify secondary causes such as eosinophilic leukemia, eosinophilia from drug reactions, and parasitic infections. Regardless of the cause of eosinophilia, EM can be life-threatening if not diagnosed early and managed appropriately.

Endomyocardial biopsy is the gold standard for diagnosis of EM [1]. Noninvasive cardiovascular imaging, however, has gradually gained more recognition as an accepted initial alternative where cardiac biopsy is not feasible. Biopsy can also be of limited sensitivity if the sample was not sufficient and imposes greater risk on the patient. It is estimated that the sensitivity of biopsy is around 54% [7]. As demonstrated by Looi et al., the use of CMR assists in the early diagnosis of the disease and the initiation of appropriate therapy [13].

In our case, the diagnosis of EM was made based on the combination of history of presenting illness, physical examination, TTE findings, and CMR along with the finding of peripheral eosinophilia which was further supported by the bone marrow biopsy. The CMR on T1-weighted fat showed increased signal intensity in the myocardium and T2 with increased signaling intensity of myocardium in the appendix of left and right ventricles consistent with myocardial hyperemia, muscular inflammation, and myocardial edema, respectively. The patient did not receive a myocardial biopsy prior to receiving treatment with steroids and had a subsequent rapid symptomatic improvement after a course of high-dose steroid therapy and was safely discharged home.

Findings that are suggestive of EM on CMR include hyperintesity on T1 (myocardial hyperemia) or T2- (tissue edema) weighted imaging with subendocardial late gadolinium enhancement and thickened fibrotic changes with inflammatory infiltrates [14, 15].

Often, there will be an associated intracardiac thrombus, which was the case in our patient as well.

Multiple limitations such as sampling errors, severe complications (perforation, tamponade), cost, and availability limit the use of endomyocardial biopsy, compromising patient safety. Therefore, a recent meeting by the International Consensus Group of Cardiovascular MR supports using CMR as a diagnosis method combined with clinical evidence [16].

Finally, another promising imaging modality is the use of PET-MRI scan as an alternative tool to assess treatment responsiveness. PET-MRI can show the gadolimium enhancement in the myopericardium and locate the inflammation [17]. However, there is no literature finding using the PET-MRI as a diagnosis tool for EM and clinical correlation, and other imaging might be necessary to support the diagnosis.

## 4. Conclusion

EM secondary to idiopathic HES remains a challenging diagnosis. The combination of clinical presentation, lab workup revealing eosinophilia, CMR findings, and TTE are helpful in the diagnosis and initiating treatment early. Findings on CMR that raise suspicion for EM include thickened fibrotic ventricles and edema of the ventricular wall.

## Data Availability

The data used to support the findings of this study are included within the article.

## Supplementary Materials

Supplementary Materials Video: transthoracic echocardiogram of parasternal long axis view showing left ventricle (LV), left atrium (LA), and increase signaling of the apex of right ventricle (RV) (red arrow).
