# Supplementary material for: Eosinophilic Myocarditis: An Often-Overlooked Diagnosis in Patients Presenting with Heart Failure
Source: Case Rep Cardiol. 2022 Jul 1;2022:8453581. doi: 10.1155/2022/8453581 (PMC9270166; doi:10.1155/2022/8453581)
Supplement: Supplementary Materials — Video: transthoracic echocardiogram of parasternal long axis view showing left ventricle (LV), left atrium (LA), and increase signaling of the apex of right ventricle (RV) (red arrow). [file 8453581.f1.zip › Supplementary file.docx]

**Supplementary file**

https://drive.google.com/file/d/14Tw3hqmeJYDPnjXeyqKZedFG5otKOcQ3/view?usp=drivesdk

Video. Transthoracic echocardiogram of parasternal long axis view showing left ventricle (LV), left atrium (LA), and increase signaling of the apex of right ventricle (RV) (red arrow).
